# Insights into VPS13 properties and function reveal a new mechanism of eukaryotic lipid transport^☆^

**DOI:** 10.1016/j.bbalip.2021.159003

**Published:** 2021-07-01

**Authors:** Marianna Leonzino, Karin M. Reinisch, Pietro De Camilli

**Affiliations:** a Department of Neuroscience, Howard Hughes Medical Institute, Program in Cellular Neuroscience, Neurodegeneration and Repair, Kavli Institute for Neuroscience, Yale School of Medicine, New Haven, USA; b Department of Cell Biology, Yale School of Medicine, New Haven, CT, USA; c Aligning Science Across Parkinson's (ASAP) Collaborative Research Network, Chevy Chase, MD, USA; d CNR Institute of Neuroscience, Milan, Italy and Humanitas Clinical and Research Center, Rozzano, MI, Italy

**Keywords:** Lipid channels, Membrane expansion, Chorein domain

## Abstract

The occurrence of protein mediated lipid transfer between intracellular membranes has been known since the late 1960's. Since these early discoveries, numerous proteins responsible for such transport, which often act at membrane contact sites, have been identified. Typically, they comprise a lipid harboring module thought to shuttle back and forth between the two adjacent bilayers. Recently, however, studies of the chorein domain protein family, which includes VPS13 and ATG2, has led to the identification of a novel mechanism of lipid transport between organelles in eukaryotic cells mediated by a rod-like protein bridge with a hydrophobic groove through which lipids can slide. This mechanism is ideally suited for bulk transport of bilayer lipids to promote membrane growth. Here we describe how studies of VPS13 led to the discovery of this new mechanism, summarize properties and known roles of VPS13 proteins, and discuss how their dysfunction may lead to disease.

## 1. Introduction

The function of eukaryotic cells is critically dependent on its compartmentalization by intracellular lipid-based membranes which have distinct lipid composition. As the backbone of most membrane lipids is synthesized in the endoplasmic reticulum (ER), and membrane lipids cannot diffuse through the aqueous environment of the cytosol, cells have evolved two major transport mechanisms to ensure the delivery of lipids to appropriate target membranes and to maintain their specific lipid composition: vesicle-based and protein-mediated lipid transport [1-3]. Vesicle-based transport, in which lipids move as part of vesicle membranes, accounts for much of membrane lipid flow within the cell, but it has important limitations. First, as delivery of ER-synthesized lipids through the classic secretory pathway involves transit through the Golgi complex, this mechanism is not adequate to replenish lipids that turn-over very rapidly in membrane regions distant from this cell compartment. For example, traffic of vesicles from the Golgi complex to nerve terminals may require many hours in long axons, given the speed of molecular motors that transport them [4]. Second, fusion/fission reactions occurring during vesicular transport are expected to results in continuous randomization of membrane lipids, as each time a vesicle fuses with another membrane, its lipids disperse in that membrane. Third, some organelles, namely mitochondria, peroxisomes, lipid droplets (and chloroplasts in plants), are not connected to the secretory pathway, yet they require ER-synthesized lipids and, conversely, they provide some lipids to other organelles [1,3]. Fourth, biochemical and structural studies, including freeze-fracture studies [5-7], have shown that the ratio between proteins and lipids in a membrane can be very variable, suggesting the need for mechanisms that control the lipid content of a membrane independently of its protein content.

Protein-mediated lipid transport is crucial to circumvent these limitations and ensures the precise tuning of lipid composition in all membrane compartments. Often these lipid transport proteins act at sites of close apposition between two membranes, so called membrane contact sites, where they may also function as membrane tethers [8]. Such localization helps make lipid transport rapid, efficient and specific. Until recently, all identified lipid transport proteins at membrane contact sites of eukaryotic cells were modular proteins comprising membrane tethering domains and a lipid transport module thought to shuttle between the two adjacent bilayers to extract and deliver lipids (“shuttle model” of lipid transfer) [9-14].

Recently, studies of VPS13 and of the autophagy factor ATG2 have suggested a completely novel form of protein-mediated lipid transport between intracellular membranes in eukaryotic cells: a flux of lipids through rod shaped lipid transport modules that act as bridges between two membranes and contain a hydrophobic groove along which lipids can slide [15-19]. Here we will focus on the properties and putative functions of the founding members of this class of lipid transport proteins, the VPS13 protein family, and on mechanisms of disease resulting from their mutations.

## 2. History

The *VPS13* gene was first identified by yeast screens aimed at uncovering genes implicated in membrane traffic at the interface between the Golgi complex and the vacuole. The name VPS13 derives from one of such screens focused on mutants with defective Vacuolar Protein Sorting [20,21]. Subsequent studies revealed *VPS13* to be the same locus as *vpt2* (from a similar screen, [21]), *soi1* (from a screen for suppressors of the mislocalization of TGN proteins induced by alteration in their localization sequence [22]), and *pep9* (from a screen for mutants defective in vacuole protease function [23]). Collectively, these studies linked Vps13 to the sorting of protein cargo destined to the vacuole and to TGN-endosome cycling of membrane proteins. However, other functions of yeast Vps13 were also identified, as this protein was found to be required for sporulation [22,24,25] and mitochondria integrity [26,27].

In 2001 loss-of-function mutations in a human gene (now called *VPS13A*) with strong similarities to yeast *VPS13* were shown to cause a very rare neurodegenerative disease called chorea-acanthocytosis [28,29]. A few years later, VPS13B mutations were reported to cause Cohen syndrome, a rare neurodevelopmental condition [30]. These findings prompted a first systematic analysis of the human VPS13 family which comprises four genes with the same domain organization and broad expression in different tissues. Primary sequence comparisons showed regions of greater conservation at the N-terminus of these proteins, referred to as chorein domain [31]. Inspection of primary sequence databases revealed that a similar domain was present in the autophagy protein ATG2, which also contains an additional stretch of similarity with VPS13 in its C-terminal region (ATG2-homology motif), suggesting a relation between ATG2 and the VPS13 protein family [31], which was recently confirmed by structural studies [18] (see below).

A major step forward in research on VPS13 function came with the discovery that in yeast dominant mutations in *VPS13* rescue phenotypes resulting from deficient function of ERMES (ER-mitochondria encounter structures) [27,32]. ERMES is a protein complex that mediates lipid transport at contacts between the ER and mitochondria [33]via lipid harboring modules of the SMP domain family present in 3 of its 4 subunits [34-36]. The same yeast studies also showed that Vps13 is localized at membrane contacts involving the vacuole and either the ER or mitochondria depending on the carbon source. These results raised the possibility that Vps13 could be involved in a path for the delivery of lipids to mitochondria which is partially redundant with the ERMES dependent path, but instead involves the vacuole as an intermediate station [27,32]. Most interestingly, the ERMES complex is not present in metazoa, suggesting the possibility that VPS13 may have a more critical function in mammals than in yeast. These findings, along with the identification of *VPS13C* as a Parkinson's disease gene [37] and of *VPS13D* as a gene whose mutations result in a spectrum of neurological disorders [38,39], triggered a renewed interest in this protein family.

Over the last few years, cell biological studies have shown that mammalian VPS13 proteins, like their yeast homologue, are localized at membrane contact sites, but with paralogue-specific localizations, suggesting duplication and specialization of *VPS13* genes during evolution [15,40-42]. Moreover, biochemical and structural studies revealed that VPS13 is indeed a protein that can harbor and transport lipids between bilayers through a novel, unconventional lipid transfer domain, and showed a similar lipid transport function for ATG2 [15-19]. These findings are summarized below.

## 3. Structure

VPS13 family proteins are distinct from previously characterized eukaryotic lipid-transporters which typically contain tea-cup like modules that transfer (through a shuttle mechanism) a single lipid [11-13,43], or at most few lipids (i.e. SMP domains [44]), at a time between organellar membranes. VPS13 proteins are big (~300–500 kDa) and elongated (~200 Å or longer), and feature a groove along their length that can host more than 10 glycerolipids. They are long enough to span between organelles at contact sites, and are proposed to act as channels that allow for efficient bulk lipid flow between organellar membranes.

**Fig. 1.**
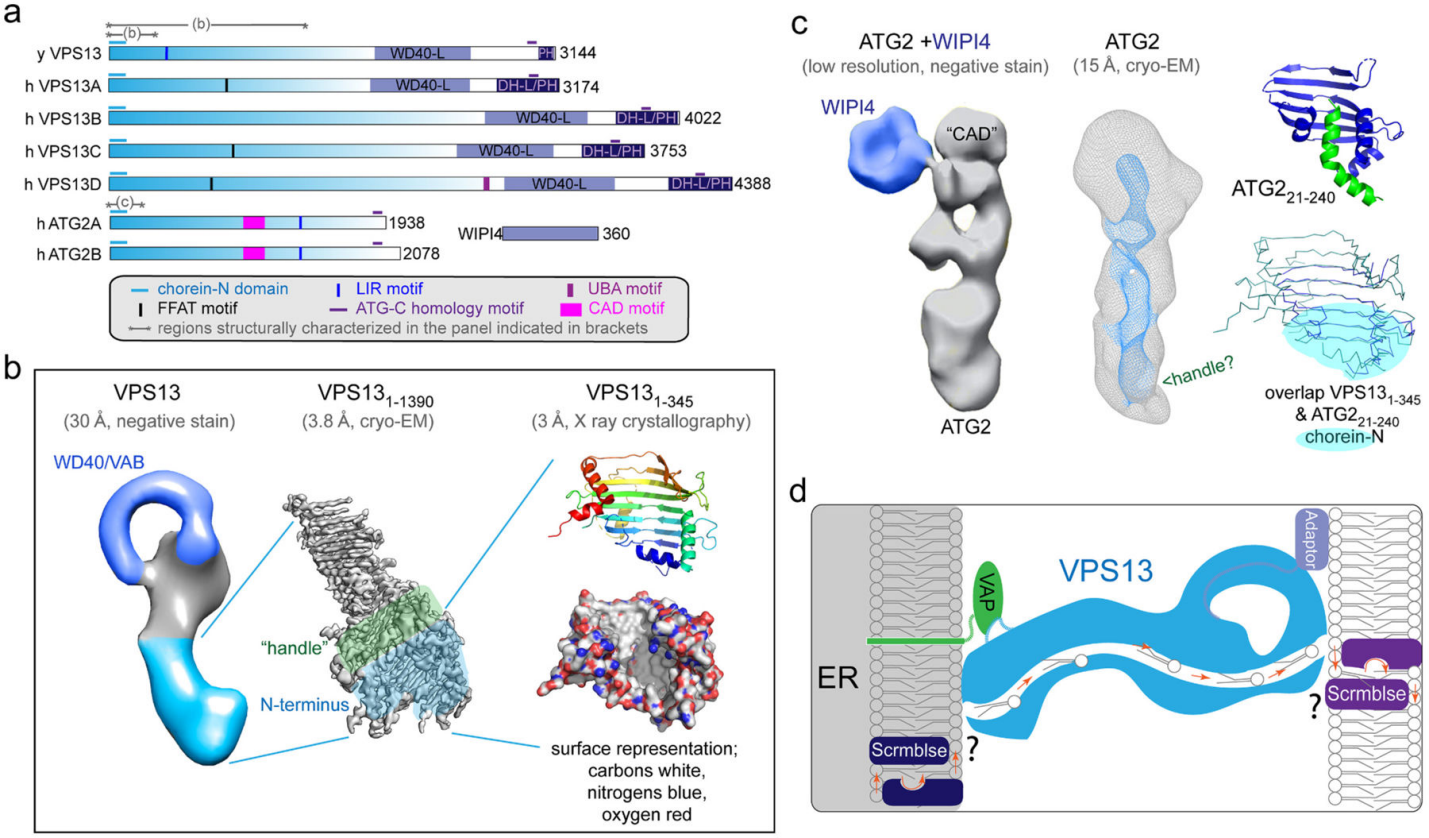
Structure and proposed function of VPS13 family proteins. (a) Domain organization of VPS13 and ATG2 proteins. (b) Structures of Vps13 and its fragments according to resolution. A negative stain reconstruction of intact VPS13 is at left [129]; the middle panel shows a larger N-terminal fragment VPS13_1–1390_ at 3.8 Å obtained by cryo-EM [16]; at right a small N-terminal fragment VPS13_1–345_ is shown in ribbons and space filling representations [15]. The latter shows that the cavity is lined with hydrophobic residues. (c) Structures of ATG2 and its fragments according to resolution. At left is a negative stain reconstruction of ATG2 bound to WIPI4 [50]; in the middle is a cryo-EM reconstruction of ATG2 alone [17]; at right is a crystal structure of an N-terminal fragment of ATG2 (residues 21–240) [18] and an overlap of this fragment and VPS13_1–345_, showing they have the same fold. The portion of ATG2 shown in green likely is rearranged in the context of the intact protein; its location in the hydrophobic groove is probably a crystallization artifact. (d) A model for bulk lipid transfer between membranes as mediated by a partnership of VPS13 family proteins with adaptors in the two membranes and by scramblases.

Sequence homology among proteins in the VPS13 family across evolution is low overall, but they share higher homology in a ~120 residue long signature domain at their N-terminus, called chorein domain (because human VPS13A is also known as “chorein”). The N-terminal fragments of Vps13 from *Chaetomium thermophilum* (residues 1–345) and of Schizosaccharomyces pombe Atg2 (residues 21–240), both including the chorein domain, have been crystallized and visualized at atomic resolution [15,18]. They form a scoop shape whose concave surface is lined by hydrophobic residues suitable for binding the hydrophobic tails of glycerolipids (Fig. 1). A larger fragment of *Chaetomium* Vps13 (residues 1–1390), visualized at 3.8 Å resolution by cryo-EM, shows that it resembles a gathering basket, including a “handle” at one end [16]. The smaller N-terminal fragment (VPS13_1–345_) forms part of the basket at the same end as the handle. The basket base is formed by a series of β-strands, whereas helices emanating from loops between the strands form the “handle”. The β-strand lined cavity defines a lipid harboring groove that extends throughout the length of the fragment (VPS13_1–1390_) and, based on bioinformatic predictions, is expected to continue further for as many as 1500 a.a. depending on the species and on the paralogue. Accordingly, it was shown that the protein can harbor at least 10 lipids [15] (see also [45]). This structure, along with the localization of VPS13 at membrane contact sites, suggests a model in which the protein can function as a bridge allowing lipids to transfer between the two apposed bilayers by sliding within its groove. Moreover, as the width of the groove is predicted to accommodate only one lipid at his narrowest point, the structure predicts unidirectional lipid transfer at any one time, resulting in net transfer of lipids from one membrane to another. The driving force for this directional flow, however, remains unknown. Downstream of this lipid transfer domain are others domains that help specify the protein's localization and cellular function. For example, all VPS13 proteins feature a β-propeller WD40-like domain followed by either a PH or a DH-like/PH unit[15] (Fig. 1). An APT1 domain has also been identified in this region [46]. Some isoform-specific features are also present, including a LIR (LC3 Interacting Region) motif in VPS13A and an UBA (Ubiquitin-Associated) Domain in VPS13D (Fig. 1a).

A low resolution cryo-EM reconstruction of full length ATG2 shows that here also the chorein domain is at one end of an elongated structure featuring a channel [17]. Thus, the stretches of primary sequence similarity in the chorein domains of VPS13 and ATG2 reflect a common architecture of their N-terminal region. ATG2 is a shorter protein that diverges from VPS13 in the C-terminal region, in spite of a short segment of primary sequence similarity close to the C-terminus of both proteins. Interestingly, ATG2 lacks the intrinsic β-propeller domain present in VPS13 proteins, but interacts with extrinsic β-propeller WD40 proteins (Atg18 in yeast, WIPI4 in metazoa) [47-49]. ATG2 also contains a CAD (Cys-Ala-Asp) motif [50] (ID: PF13329) (Fig. 1).

Protein mediated lipid transfer occurs exclusively between the cytosolic, and not luminal, leaflets of apposed organellar membranes at membrane contact sites. Directional bulk lipid transfer, as proposed for VPS13 family proteins, would then result in bilayer asymmetry both in the membrane from which lipids are depleted as well as in the membrane to which they are added, unless there are mechanisms that reequilibrate the leaflets in these membranes. Strong support for the occurrence of such mechanism comes from recent studies of ATG2, which localizes to ER-autophagosome contact sites and whose lipid transfer ability is required for the formation of the autophagosomal membrane [17]. These studies show that ATG2 interacts physically with ATG9 [51,52], an integral membrane protein of Golgi-derived vesicles long known to play a role in the initiation and expansion of the isolation membrane of autophagosomes, and that ATG9 is a lipid scramblase [52-54]. Additionally, a recent study indicates that two ER resident protein known to be required for autophagosome growth, TMEM41B and VMP1 [55-57], are also scramblases and interact with the chorein domain of ATG2 [58]. Plausibly, VPS13 and other chorein family proteins also function in cooperation with scramblases. In fact, VMP1 was recently reported to act upstream of VPS13D in a process leading to mitophagy in flies [59]. Moreover, it was shown that human VPS13A interacts with XK [60], a paralogue of proteins with scrambling activity [61] (see below).

While until recently there was no evidence for a bridge-like model of lipid transport between bilayers in eukaryotic cells, the occurrence of proteins or protein complexes that mediate membrane lipid transfer between bilayers is known to occur in gram-negative bacteria, where they transfer lipids between the bacterial inner and outer membranes. Intriguingly, these protein machineries also incorporate lipid transfer bridges comprising mostly β-strands, reminiscent of VPS13 [62-64] as well as multispan integral membrane components in both membranes.

## 4. Sites of action

### 4.1. Yeast

In yeast, the single Vps13 acts at multiple cellular sites, with differences depending on the carbon sources available and thus on the metabolic state. When cells grow in the presence of glucose, Vps13 localizes at the interface between mitochondria and the vacuole (the yeast lysosome) and on endosomes. In non-fermentative conditions, Vps13 is instead recruited to the nuclear-vacuole junction, a contact between the nuclear envelope, which is a specialized region of the ER, and the vacuole [27,32]. In addition, during meiotic division Vps13 binds to the prospore membrane [24,25]. Adaptor proteins for Vps13 on mitochondria, the vacuole and the prospore membrane have been identified as Mcp1, Ypt35 and Spo71, respectively [25,65,66]. Interestingly, all these proteins bind the β-propeller/WD40-like region of Vps13, hence named VAB (for Vps13 Adaptor Binding) domain, via a conserved proline-X-proline motif [66]. The competitive nature of these interactions speaks against a model in which the localization of Vps13 at vacuole-mitochondria contacts involves its simultaneous binding to Mcp1 (on the outer mitochondrial membrane) and to Ypt35 (on the vacuole). As studies of mammalian VPS13 proteins (see below) suggest that a shared property of VPS13 may be to bridge the ER to other membranes, an intriguing possibility requiring future study is that Vps13-positive mitochondria-vacuole contacts detected by fluorescence may include an ER cisterna making contact with both organelles. Supporting this possibility, it was shown that Vps13-positive contacts between the vacuole and mitochondria are distinct from the so-called vCLAMP contacts, known to be bona fide direct interactions between the two organelles [67].

### 4.2. Mammalian cells

**Fig. 2.**
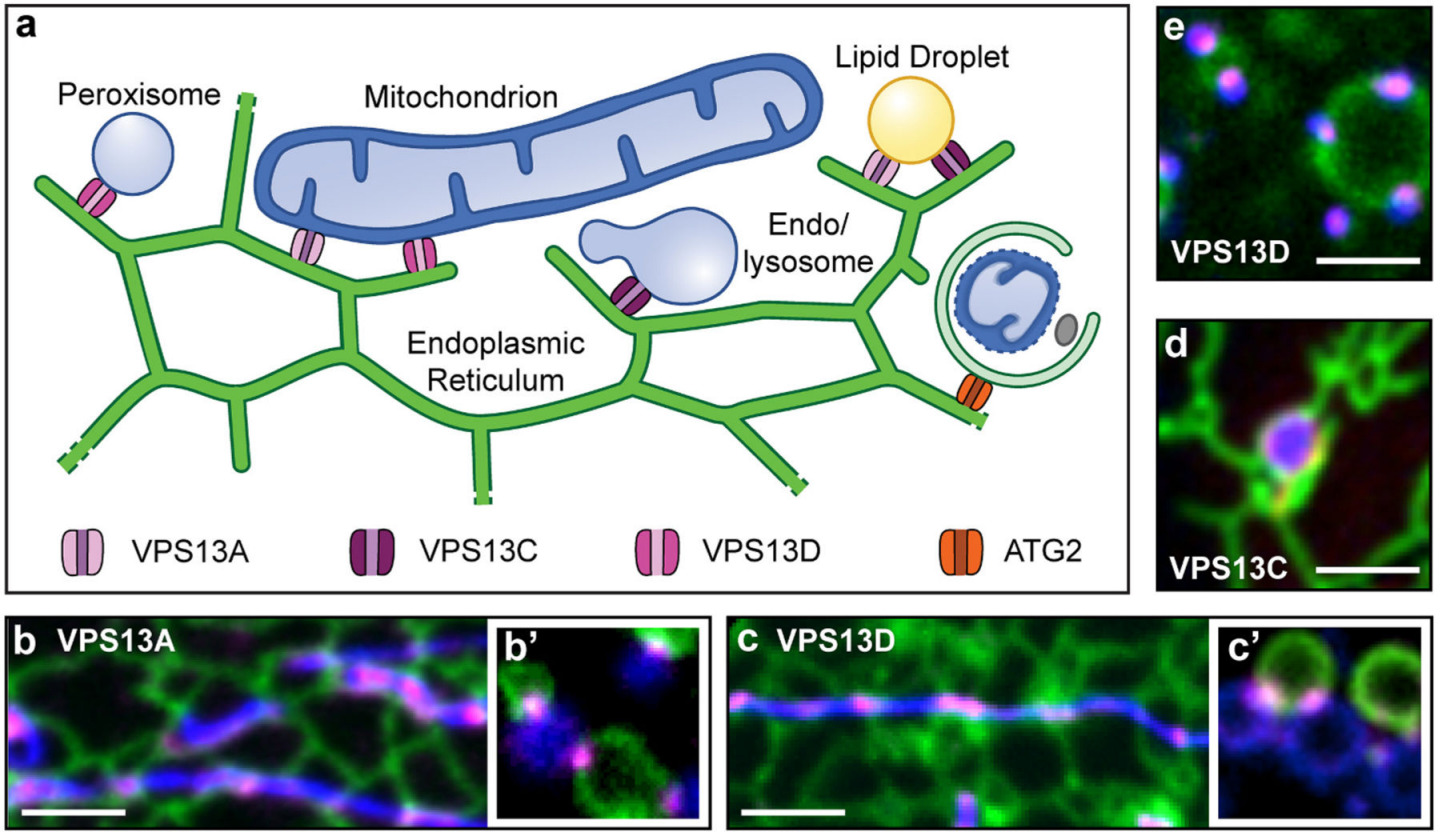
Mammalian VPS13 proteins localize at membrane contact sites. (a) Cartoon depicting the localization of VPS13A, VPS13C, VPS13D and ATG2 at contact sites between the ER and other organelles. Fluorescence panels (b to e) are snapshots from live cell imaging experiments showing the contacts indicated with the corresponding letter in the main panel. In each image the ER marker is in green, the specific VPS13 protein is in magenta and the tethered organelle is in blue: mitochondria in b and b′ and c and c′, an endolysosomes in d and peroxisomes in e. Panels b′, c′ and e are from cells exposed to hypotonic shock. Such treatment induces vesiculation of organelles [130] which remain connected by tethering proteins, allowing a better visualization of VPS13 localization at organelle contact sites. Micrographs of b, c, c′ and d were cropped from data published in [15,40]. Micrographs b′ and e are unpublished data (panel b′ from M. Leonzino and panel e from M. Leonzino and A. Guillén-Samander).

The *VPS13* gene underwent successive duplications during evolution. The information already acquired on VPS13 proteins of higher eukaryotes indicates that the multiple and distinct functions of yeast Vps13 have at least in part segregated into the multiple VPS13 paralogues. Two *VPS13* genes are found in worms, one corresponding to *VPS13A/C* and the other to *VPS13D*. Flies express three VPS13 paralogues, with two of them representing orthologues of mammalian VPS13B and VPS13D respectively, while the third is most similar to mammalian VPS13A and VPS13C [84,132]. Mammalian VPS13A and VPS13C most likely are the result of an additional gene duplication, and, accordingly, are the most similar to each other within the VPS13 family [31]. As shown for yeast Vps13, most localization data on these proteins, corroborated by the identification of binding partners on two different organelles, suggests an action at membrane contact sites [15,41,42,68]. Most evidence for mammalian VPS13A, VPS13C and VPS13D indicates that they specifically localize at contacts between the ER and other organelles [15,40-42] (Fig. 2). Hence (see also above) it is possible that the ER may be a shared component of all VPS13 containing membrane contact sites (but see ref. [45,69] for reported localizations of VPS13D at lipid droplet/mitochondria contacts and of VPS13B at endosome-endosome contacts, respectively). Binding of these three proteins to the ER is mediated by VAP[15] [40], a small tail-anchored protein of the ER membrane with a cytosolic “MSP” domain that binds proteins containing the so-called FFAT motif [15] [40]. VPS13A and VPS13C bind VAP through a canonical FFAT motif [15,70], while VPS13D binds VAP through a recently described modified version of the FFAT motif, referred to as phospho-FFAT motif [40,71].

Both VPS13A and VPS13D bridge the ER to mitochondria (Fig. 2), but through different interactions at the mitochondrial membrane. Moreover, neither of these interactions is conserved from yeast, as MCP1, the outer mitochondrial protein that binds Vps13 in this organism [65], is not present in higher eukaryotes. VPS13A binds unidentified binding site(s) on the mitochondria via its C-terminal DH-PH region [15]. In contrast, VPS13D binds the outer mitochondrial membrane GTPase Miro (both Miro1 and Miro2) likely via the β-propeller/WD40-like/VAB domain [40]. Interestingly, the yeast orthologue of Miro, Gem1, is also thought to have a regulatory role in lipid transport between the ER and mitochondria as it is an accessory subunit of the ERMES complex [72,73]. However, in metazoa, where ERMES is not present, Miro was until now known primarily for its role in mitochondrial motility [74-79]. Since a splice variant of Miro1 is partially targeted to peroxisomes [80-82], a pool of VPS13D also bridges the ER to peroxisomes [40] (Fig. 2). VPS13D was also reported to function at the interface between mitochondria and lipid droplets, but in this case an N-terminal portion of VPS13D was implicated in mitochondria tethering [45], suggesting a yet different mechanism of interaction with mitochondria that needs further investigation.

An additional pool of VPS13D is localized in the Golgi complex, where it could function as a VAP-dependent tether between the ER and the Golgi membranes [40]. So far, however, a binding partner for VPS13D in the Golgi complex has not been identified.

Despite its close similarity to VPS13A (including in the DH-like/PH region that mediates binding of VPS13A to mitochondria), VPS13C has a different localization. Fluorescence imaging of both endogenous and overexpressed VPS13C revealed that it bridges the ER to late endosomes and lysosomes (Fig. 2) via its β-propeller/WD40-like/VAB region [15]. These data were subsequently supported by experiments that identified VPS13C, but not VPS13A and D, as an effector of late endosomal Rabs (Rab7) [83] and as a neighbor of lysosomes in proximity biotinylations studies [41,49]. The interaction of VPS13C with endo/lysosomes likely reflects a conservation of the interaction of yeast Vps13 with the yeast vacuole, although mechanistic aspects of the interactions are different as in yeast they involve a sorting nexin (Ypt35) instead of Rabs [66]. An endosomal localization (based on subcellular fractionation) was also proposed for Drosophila Vps13, an orthologue of mammalian VPS13A and VPS13C, suggesting that duplication of this gene in mammals resulted in divergence of function [84]. Finally, the role of VPS13 family proteins in the phagocytosis in some unicellular organisms, is consistent with an action of VPS13 isoforms in the endocytic pathway [85,86].

Both VPS13A and VPS13C [15,41], as well as VPS13D [45], when overexpressed, bind lipid droplets through an interaction involving an amphipatic helix in their DH-like/PH domain regions and localize at ER-(VPS13A and VPS13C) or mitochondria- (VPS13D only) contacts with them. At least in the case of VPS13C this localization is consistent with its enrichment in brown adipose tissue [87] and its reported role in adipogenesis [88].

An organelle bridging function for VPS13B remains elusive. Exogenously expressed VPS13B localizes to the Golgi complex, at least in part via an interaction mediated by RAB6 [69,89,90]. Whether this localization reflects a function of VPS13B at ER-Golgi complex contacts, possibly redundant with the function of VPS13D at this site (see above), remains to be determined, as no binding of VPS13B to VAP or to the ER has yet been detected. A role for VPS13B in endosome tethering has also been reported [69].

## 5. A putative function in membrane growth and expansion

The channel model of VPS13 and ATG2 action suggests a role in membrane growth and expansion. Thus, it is of interest to consider whether the sites of actions of these proteins, and the phenotypes resulting from their absence, are compatible with such function.

**Fig. 3.**
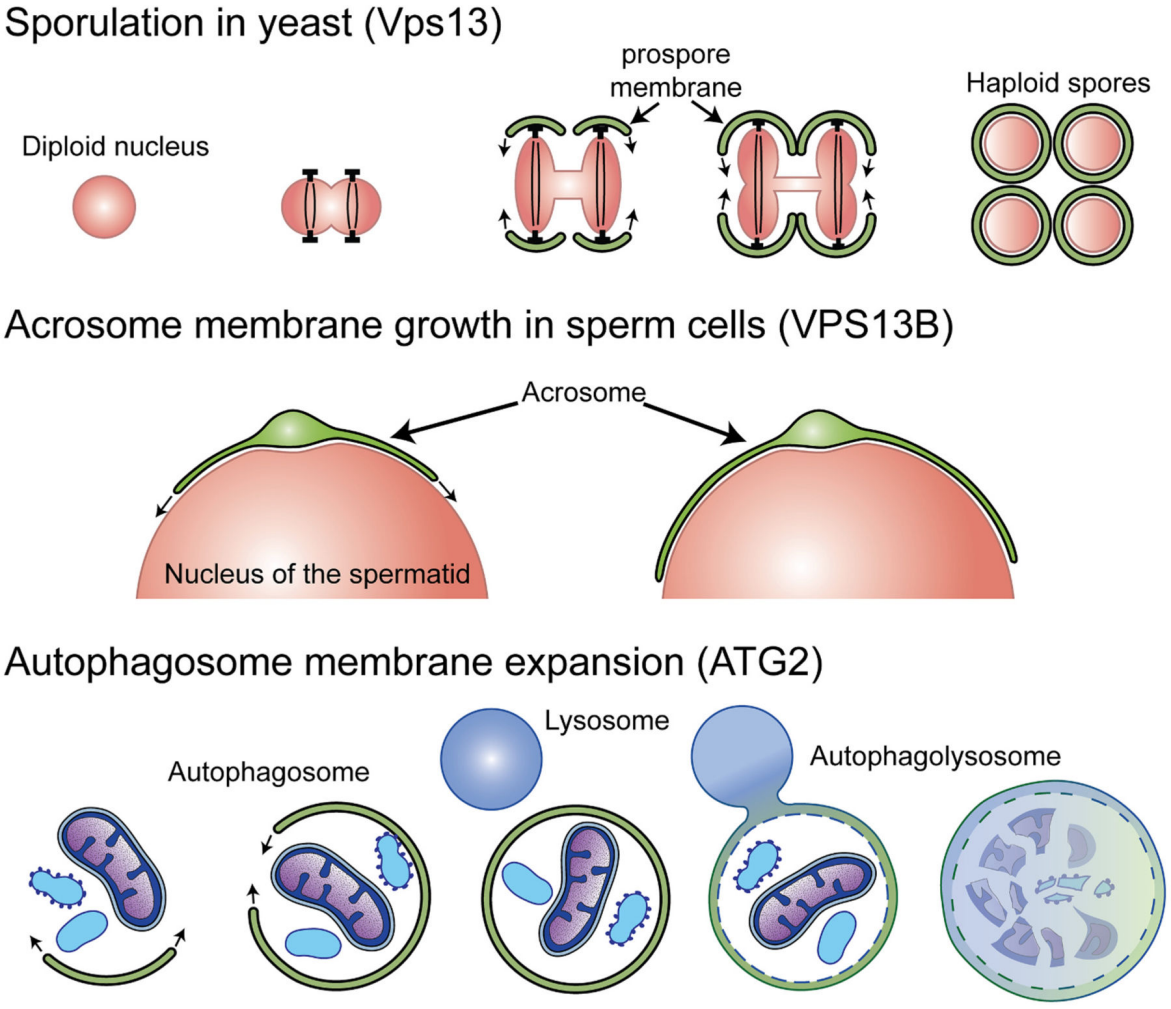
VPS13 and ATG2 are required for membrane expansion. The upper section depicts the process of sporulation in yeast, during which the prospore membrane grows around each of the haploid nuclei generated by meiosis. Expansion of the prospore membrane requires Vps13 [24]. The middle section depicts acrosome formation in mammalian spermatids. This process requires VPS13B [91]. The lower section shows the process of autophagy where ATG2 is needed for the growth of the isolation membrane [17,18,131]. Once the autophagosome is closed, fusion with a lysosome ensures degradation of its content.

At least three cases can be pointed out, where genetic data clearly support such a mode of action, with a unidirectional flow of lipids into expanding membranes (Fig. 3). One is yeast sporulation. During the second meiotic division, new membranous cisternae are generated at the 4 spindle poles. These cisternae will eventually encapsulate the 4 new haploid nuclei and become the precursors of the membranes that encapsulate the spores (Fig. 3, upper panel). During this process, Vps13 is localized on the sporulation membrane and VPS13, including its hydrophobic channel, is required for its growth [16,24]. Another case is the growth of the acrosomal membrane of spermatocytes during the second meiotic division in mammals, a process that likely has evolutionary relation to yeast sporulation. VPS13B, which is localized in the Golgi complex, is required for the growth of the acrosomal membrane [91], which like the yeast sporulation membrane starts as a membranous cisterna that grows in proximity of the haploid nucleus (Fig. 3, middle panel). The third case is the role of ATG2 in the growth of the isolation membranes of the autophagosome [92,93] (Fig. 3, lower panel). Until recently, the source of phospholipid for such membrane had remained unclear. However, several recent studies have provided strong evidence for a model in which ATG2 functions as bridge through which bilayer lipids from the ER can flow to the isolation membrane to mediate its expansion [17-19]. The low abundance of intramembranous particles visible by freeze-fracture in autophagosome membranes [6,94]and in the acrosomal membrane [95]is consistent with a model according to which such membranes grow primarily by delivery of membrane lipids.

Bulk delivery of phospholipids could also underlie the function of VPS13 proteins at contacts between the ER and either mitochondria or peroxisomes, as these organelles do not receive ER-synthesized lipids via vesicular transport. Since most of the membrane lipids of these organelles are derived from the ER, there should be a mechanism to account for net flux of lipids from the ER membrane. VPS13 proteins are ideally suited for such function. In cells of metazoa, the role of VPS13 at mitochondria (i.e. the collective function of VPS13A and VPS13D) may be particularly important as ERMES is not present in these species. While the absence of VPS13A does not result in major cellular phenotypes (our unpublished observation and [96,97]), and is compatible with life in humans [28,29], the absence of VPS13D is incompatible for cellular and organismal life [38,98-100], suggesting a critical importance of VPS13-dependent ER-mitochondrial junctions in mammalian cells. Moreover, genetic defects that impair, but do not abolish, VPS13D function in both Drosophila and mammalian cells result in abnormal swollen mitochondria, which eventually undergo rupture leading to leakage of their content, including DNA [38,100] [101]. Consistent with these results, loss-of-function alleles in yeast *VPS13*, were identified in a screen for genes whose disruption results in mitochondrial DNA leakage [24,26,102]. One potential, albeit speculative, interpretation of these finding is that mitochondria swelling may reflect an increased volume to surface ratio due to deficient membrane growth. In a striking confirmation of the hypothesis that VPS13D functions at ER-peroxisome contacts to provide the membrane lipids needed for peroxisome growth, a defect in peroxisome biogenesis was observed in VPS13D deficient cells [103].

How VPS13-mediated bulk transfer of lipids may be implicated in membrane traffic between the Golgi and endosomes and in endo/lysosomal function, as suggested by yeast screens and by the localizations of certain VPS13 paralogues, remain questions for future studies. In yeast, the defect in sorting of carboxypeptidase Y (CPY) from Golgi to the vacuole is the robust phenotype that gave the name to the protein [20,21]. One possibility is that bulk delivery of bilayer lipids from the ER to membranes that participate in these transport reactions is required to control their proper protein/lipid ratio with implications for normal vesicle traffic and protein sorting fidelity. However, other hypotheses can be considered, including a potential role of VPS13 in the delivery specific lipid species.

In the case of lipid droplets, VPS13A- and VPS13C-dependent lipid transport from the ER to their outer monolayer may play a role in their expansion. Accordingly, VPS13C was implicated in adipogenesis [88]. However, the study that reported presence of VPS13D at mitochondria-lipid droplets contacts proposed a role of VPS13D in the transport of fatty acids from lipid droplets to mitochondria upon starvation [45]. Such role would imply an opposite direction of lipid transport, “from” rather than “to”, lipid droplets. Future studies will be required to further elucidate the role of different VPS13 isoform in lipid droplet biology.

## 6. Disease mechanisms

Studies of diseases resulting from defects in VPS13 family proteins had suggested a variety of pathogenic mechanisms. The recently discovered lipid transport function of VPS13 family proteins, and more specifically their likely role in bulk lipid transfer, supports the idea that phenotypic manifestations are the results of a primary defect in intracellular lipid homeostasis/transport. The distinct clinical manifestations of defects in each of the VPS13 paralogues may be the consequence of their specialized properties, such as of their different intracellular sites of action. Alternatively, or in addition, they may be the consequence of their different levels of expression in distinct cell populations, although inspection of databases suggests a broad expression of all four paralogues in most tissues.

### 6.1. VPS13A

Homozygous null mutations in *VPS13A* result in chorea-acanthocytosis, an age-dependent Huntington chorea-like neurodegenerative disease characterized by progressive loss of movement control and cognitive functions (hence the name Chorein for VPS13A) [28,29]. The disease is accompanied by the presence of abnormally-shaped red blood cells, referred to as acanthocytes (irregularly shaped red cells with spurs, also called spur cells). The neurologic effects are caused by degeneration of striatal neurons, possibly due to impaired mitochondrial function in view of the site of action of the protein, but the reason for the selective impairment of this neuronal population is not known. In VPS13A-KO mice, phenotypic manifesations reminiscent of those of human patients have a variable penetrance, depending on the background strain [104,105]. However, an interesting phenotype observed in this model is a striking alteration of mitochondrial morphology in the sperm midpiece, which leads to their infertility [106]. This finding underscores the importance of VPS13A for mitochondrial function.

The core neurological and hematological defects of chorea-acanthocytosis, are shared with three other conditions, collectively named Neuroacanthocytosis [107]. Interestingly, two such diseases (McLeod syndrome and PKAN) are caused by defects in proteins involved in membrane lipid dynamics.

McLeod syndrome, which more closely resembles chorea-acanthocytosis [108] is due to mutations of the *XK* gene [109] which encodes a protein enriched in the erythrocyte membrane (X-Linked Kx Blood Group) [60]. As discussed above, the predicted function of VPS13 proteins in phosholipid transport is thought to require the action of lipid scramblases to allow for equilibration of phospholipids between the bilayers of donor and acceptor membranes. It is therefore of interest that XK is a paralogue of proteins with scrambling activity, although a scrambling function for XK itself remains to be proven [61]. Notably, cell-based overexpression studies have suggested a physical interaction between XK and VPS13A [60] and, consistently, reduced levels of VPS13A have been reported in McLeod patients [110]. However, as the bulk of XK is thought to be primarily localized at the plasma membrane, the functional connection between these two proteins remains to be further explored. Likewise, how a deficiency in VPS13A results in acanthocytosis remains poorly understood [111], as mature mammalian erythrocytes lack internal membranes.

PKAN is due to mutations in the gene encoding panthotenate kinase 2 (*PANK2*), the rate-limiting step in the biosynthesis of CoA, which is a cofactor in the synthesis of fatty acid [112]. Since this enzyme is localized in mitochondria, the similar pathological manifestations of PKAN and chorea-acanthocytosis may be related to the importance of both PANK2 and VPS13A in aspects mitochondrial physiology dependent on normal lipid homeostasis.

### 6.2. VPS13B (COH1)

Recessive loss-of-function mutations in the gene encoding VPS13B are responsible for Cohen syndrome (hence the alias COH1), a neurodevelopmental condition characterized by heterogeneous manifestations including microcephaly, intellectual disabilities and hypotonia [30]. Given the concentration of the protein in the Golgi complex [69,89,90], disease likely results from a disruption of Golgi membrane dynamics, leading to abnormal membrane traffic, or from alterations in the biochemical reactions that occur in this cell region. An example of these defects is the impaired formation of the acrosome in spermatocytes, as discussed above [91]. Another example is the abnormal glycosylation state of some circulating proteins [113]. However, much remains to be understood about VPS13B-dependent disease mechanisms.

### 6.3. VPS13C

Loss-of-function mutations of VPS13C result in early onset Parkinson's disease (hence the PARK23 designation of this protein) [37,114,115], while partial loss-of-function increases Parkinson's disease risk [116,117]. Loss-of-function VPS13C mutations have also been reported in patients with Lewy Bodies dementias with or without Parkinsonism [118,119]. The link of VPS13C to Parkinson was discovered shortly after yeast Vps13 was shown to be important for mitochondrial integrity. Hence it was proposed, with the support of biochemical data, that VPS13C plays a direct role in mitochondrial physiology [37]. The subsequent discovery that VPS13C is localized at contacts between the ER and late endosomes/lysosomes calls for a revision of this hypothesis [15,134]. Interestingly, while several Parkinson's disease genes encode proteins that are directly implicated in mitochondria quality control mechanisms (such as Pink1 and Parkin) [120], many other such genes encode proteins implicated in endosomal or lysosomal function [121,122]. An indirect effect of alterations in lysosomal lipid homeostasis on mitochondrial health could be speculated, as many recent studies have reported mitochondrial damage induced by lysosomal dysfunction via an interplay mediated by contact sites [123], metabolic pathways [124], as well as mitophagy [125]. Supporting this possibility a recent study provided evidence for the an activation of the STING-cGAS innate immune pathway in VPS13C KO cells, which is dependent both on mitochondrial DNA leakage and defective degradation of activated STING in lysosomes [134].

### 6.4. VPS13D

As mentioned above, *VPS13D* appears to be an essential gene, and complete absence of VPS13D is incompatible with life. Both mice (MGI2448530) and flies [38,100] lacking VPS13D die during embryonic development and all patients carrying VPS13D mutations appear to have at least one partially functional allele [38,39]. Indeed, no patients with homozygous loss-of-function mutations in VPS13D have been found. VPS13D biallelic mutations lead to early-onset, heterogeneous movement disorders, including ataxias. The severity of the disease likely depends on the degree of residual function of the mutated VPS13D protein [38, 39]. In light of the cell biological findings discussed above, mitochondria and peroxisome dysfunctions are likely to play a major role in these conditions. The implication of mitochondrial dysfunctions in the pathogenesis of the conditions arising from VPS13D malfunctions are also supported by studies in Drosophila showing severe mitochondrial alterations and defects in mitophagy upon VPS13D down-regulation [38,59,100,101]. Additional defects resulting from the pool of VPD13D localized in the Golgi complex area should also be considered.

## 7. Concluding remarks

The characterization of the VPS13 protein family and of the related protein ATG2 has introduced a new concept in cell biology: the net flow of bilayer lipids between membranes through molecular pipelines independent of classic vesicular transport. While a “pipeline-based” mechanism for lipid transfer had been described in bacteria [126], there was no clear evidence of its existence in eukaryotic cells. This mode of lipid transport by VPS13 and ATG2 needs to be further validated, but is already grounded in genetic, imaging, structural and biochemical data. Such mechanism can account for the rapid growth of new membranes and for the delivery of lipids to membranous organelles that are not connected to the secretory pathway such as mitochondria and peroxisomes. Much remains to be learned about the precise function and lipid transport properties of VPS13. In this review we have favored the idea that VPS13 acts at the interface of the ER with other membranes with a flow of lipids out from the ER. However, other possibilities should also be considered in view of reported localizations of VPS13 family members at sites that do not appear to involve ER [27,32,45,69]. The driving force(s) responsible for lipid transport remain to be elucidated. These may include passive mechanisms driven by physical forces such as different internal pressure between donor and acceptor organelles or ATP-driven mechanisms, as shown for the transport of lipopolysaccharide between the inner and outer membranes of GRAM-negtaive bacteria [126]. Preferences for specific lipids remain to be further investigated, and the mechanisms by which transport is regulated remain unclear. Investigations of splice variants of VPS13 paralogues may reveal yet unknown sites of actions of these proteins. Inspection of databases reveal other proteins with distant similarities to VPS13 and ATG2, suggesting that chorein-domain proteins may have a more widespread role in cell function than currently appreciated. Finally, functional overlaps of these various proteins, including between VPS13 and ATG2, should also be considered as VPS13 family proteins have been implicated in autophagy [96], mitophagy [59,100,101] and ER phagy [127], while yeast ATG2 was reported to be required for sporulation, a VPS13-dependent process [128].

Finally, further elucidating the function of each VPS13 paralogue will be important to develop specific therapies for diseases resulting from their mutations. Conversely, studies of such diseases will provide further insight into the function of the VPS13 protein family, providing a striking example of the synergy of human medicine and fundamental cell biology in advancing our understanding of basic mechanisms of life.
